# Virological and immunological failure of HAART and associated risk factors among adults and adolescents in the Tigray region of Northern Ethiopia

**DOI:** 10.1371/journal.pone.0196259

**Published:** 2018-05-01

**Authors:** Genet Gebrehiwet Hailu, Dawit Gebregziabher Hagos, Amlsha Kahsay Hagos, Araya Gebreyesus Wasihun, Tsehaye Asmelash Dejene

**Affiliations:** 1 College of Health Sciences, Department of Medical Microbiology and Immunology, Mekelle University, Mekelle, Tigray, Ethiopia; 2 College of Health Sciences, Ayder Comprehensive Specialized Hospital, Department of Laboratory Mekelle University, Mekelle, Tigray, Ethiopia; 3 College of Health Sciences, Department of Medical Microbiology, Axum University, Tigray, Ethiopia; National and Kapodistrian University of Athens, GREECE

## Abstract

**Background:**

Human immunodeficiency virus/Acquired immunodeficiency syndrome associated morbidity and mortality has reduced significantly since the introduction of highly active antiretroviral therapy. As a result of increasing access to highly active antiretroviral therapy, the survival and quality of life of the patients has significantly improved globally. Despite this promising result, regular monitoring of people on antiretroviral therapy is recommended to ensure whether there is an effective treatment response or not. This study was designed to assess virological and immunological failure of highly active antiretroviral therapy users among adults and adolescents in the Tigray region of Northern Ethiopia, where scanty data are available.

**Methods:**

A retrospective follow up study was conducted from September 1 to December 30, 2016 to assess the magnitude and factors associated with virological and immunological failure among 260 adults and adolescents highly active antiretroviral therapy users who started first line ART between January 1, 2008 to March 1, 2016. A standardized questionnaire was used to collect socio-demographic and clinical data. SPSS Version21 statistical software was used for analysis. Bivariate and multivariate logistic regression analyses were conducted to identify factors associated to virological and immunological failure. Statistical association was declared significant if p-value was ≤ 0.05.

**Result:**

A total of 30 (11.5%) and 17 (6.5%) participants experienced virological and immunological failure respectively in a median time of 36 months of highly active antiretroviral therapy. Virological failure was associated with non-adherence to medications, aged < 40 years old, having CD4^+^ T-cells count < 250 cells/μL and male gender. Similarly, immunological failure was associated with non-adherence, tuberculosis co-infection and Human immunodeficiency virus RNA ≥1000 copies/mL.

**Conclusions:**

The current result shows that immunological and virological failure is a problem in a setting where highly active antiretroviral therapy has been largely scale up. The problem is more in patients with poor adherence. This will in turn affect the global targets of 90% viral suppression by 2020. This may indicate the need for more investment and commitment to improving patient adherence in the study area.

## Introduction

Though, there is no curative therapy for Human immunodeficiency virus (HIV), the introduction of highly active antiretroviral therapy (HAART) in the late 1990s has substantially decreased HIV associated morbidity and mortality, and increased patient survival [1–3]. Proper use of these medications have transformed the disease from an inevitably fatal disease to a chronic, treatable condition by suppressing the viral load to undetectable levels and providing a consistent increase in the number of CD4^+^ T lymphocytes [4,5]

Major challenges especially in low and middle income countries such as poor adherence to medications [6], defects in host immunity, development of drug resistance [7,8], toxicity, poor infrastructure and lack of specialized care and human resources [9] are factors hindering the success of HAART. Furthermore, there were more debates on the introduction of HAART particularly to sub-Saharan Africa mainly due to adherence concerns and subsequent development of drug resistance [10]. Even with these limitations, HAART has been significantly scaled up at global level as per the World Health Organization (WHO) recommendations [11].

HIV infected individuals on HAART are usually recommended to monitor clinically, immunologically and virologically on a regular manner to ensure successful treatment, identify adherence problems and determine whether HAART regimens should be switched or not. Compared to clinical or immunological monitoring, virological monitoring provides an early and more accurate indication of treatment failure and the need to switch from first-line to second-line drugs, reduce the accumulation of drug resistance mutations and improving clinical outcomes. However, due to the high costs and technical demands of the test, clinical and immunological assessments are usually recommended to start and monitor the efficacy of therapy [12]. As a result, patients may be at risk of unrecognized virologic failure, subsequent development of drug resistance and to continue with failed regimen.

As of 2016, 19.5 million people living with HIV were accessing ART with an approximate coverage of 53%. Of these, 11.7 million people accessing ART were from East and southern Africa with coverage of 60% [13]. Ethiopia is one of the sub-Saharan African countries with high burden of HIV infection, estimated adult prevalence 1.1% (0.8% in males and 1.5% in females) and 0.03% incidence. In Ethiopia HAART began in 2003 and scale up in 2005. Since then, a total of 375,811 people living with HIV (PLWH) were actively on HAART by 2015 with 82.8% ART coverage [14], and improved health and survival of patients in many parts of Ethiopia [15].

There are substantial numbers of ART users in the Tigray regional state [16], however, there is no data on the immunological and virological outcomes of HAART. Hence, addressing this knowledge gap is reasonable to help public health planners, policy makers and implementers to plan and design appropriate intervention strategies in order to prevent the disease.

## Materials and methods

### Study area and sample size determination

The study was conducted in Mekelle Hospital (MH) and Ayder Comprehensive Specialized Hospital (ACSH), both located in Mekelle city. Mekelle, the capital city of Tigray Regional state is located 783 Km north of Addis Ababa, the capital city of Ethiopia. There are five public hospitals in the city. These two hospitals were selected purposely due to their higher flow of patients. MH is administered by the Tigray Regional Health Bureau, whereas ACSH is a University hospital under the Federal Ministry of Education. According to the information obtained from the ART clinics, by May 2016 there were about 8,727 and 1,564 people living with HIV (PLHIV) ever enrolled; 6,789 and 1,387 ever started ART; 4,189 and 1,222 were actively on ART in MH and ACSH, respectively. The sample size (n = 260) was determined using the single mean proportion formula.

#### Study design and data collection procedures

A retrospective follow up study was used on HIV infected individuals on first line ART. Base line and follow up clinical and laboratory data were collected from each study participants’ medical records using checklists. In addition, socio demographic characteristics, clinical and laboratory data (viral load and CD4^+^ count) were also collected from each study participants prospectively using questionnaire during data collection period, September 1 to December 30, 2016.

#### Study population and study variables

The study population were adolescents (aged 10–19) and adults (≥20 years) who initiated HAART between January 1, 2008 and March 1, 2016. Patients who were less than 6 months on ART, who had been on second line regimen, lost to follow up, febrile, and transfer in were excluded from the study. Virological and immunological failures defined based on WHO consolidated guide line 2016 were the primary outcome variables of the study. Socio-demographic characteristics (age, gender, educational level, occupation, marital status and socio economic status) and information related to HIV infection and ART (date of ART initiation, route of transmission, time on ART, HIV prophylaxis given, presence/absence of opportunistic infections, nutritional status, types of drugs and regimen given, HBV and HCV co-infections, level of adherence, WHO clinical stage, and known chronic non communicable diseases) were the predictor variables.

#### Laboratory testing methods

**RNA extraction and Plasma Viral load Determination**: During the collection time we collected blood samples for each patient and did a single viral load using the standard procedure. Briefly, HIV-1 RNA was extracted from 0.2 mL of plasma using Abbott *m2000sp* automated sample preparation system (Abbott Molecular, USA) in Tigray Health Research Institution (THRI). Extracted RNAs were measured using Abbott *m2000rt* quantitative Real Time HIV-1 assay (Abbott Molecular, USA) with HIV-1 RNA detection level of 40 to 10 million copies/mL based on the manufacturer’s procedures.

**Fig 1:**
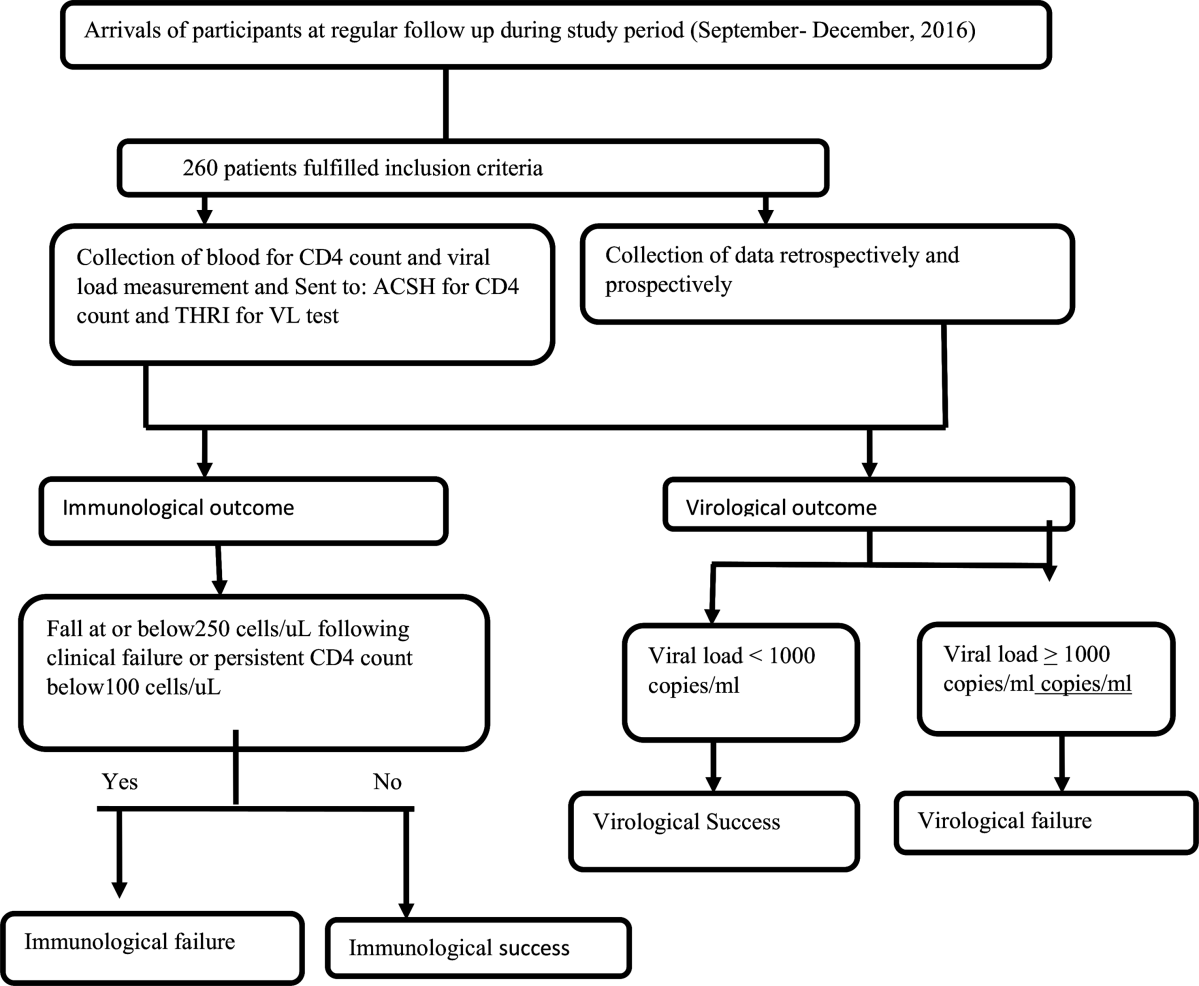
Work flow of the study.

**CD4**^**+**^ **count:** Data on CD4^+^ T-cells count which was measured at baseline and every six months of interval then after during regular follow up visit was collected from medical charts. In addition, we have also collected blood samples from each participant during the data collection period and measured CD4+ count at ACSH ART laboratory using the BD FACSCount ^TM^ (Becton Dickinson, USA) following the manufacturer’s protocol (“**Fig 1”)**.

#### Data quality assurance, processing and analysis

Quality of laboratory data was kept by strictly following standard operational procedures during sample collection, handling and transportation. Negative, low positive and high-positive (during viral load determination); and low, medium and high controls (during CD4^+^ count) were included in each test to evaluate run validity. Furthermore, CD4^+^ and viral load reagents were checked for expiry date. Filled forms and questionnaires were checked for completeness and consistency daily during data collection period by principal investigator.

Data was entered and validated using Epidata version 3.1 and exported to SPSS version 22 for statistical analyses. Descriptive statistics, such as median and inter quartile ranges (IQR) was used to compute continuous variables and counts with percentage for categorical variables. Binary and multiple logistic regressions were used to examine the relationships of independent variables with the primary outcomes. A p-value of ≤ 0.05 with 95% confidence interval was considered as statistically significant correspondingly.

#### Ethical considerations

Institutional ethical clearance was obtained from the Mekelle University, College of Health Sciences Ethical Review Committee (ERC0816/2016). Written informed consent or assent was also obtained from study participants and/or families and/or guardians. Furthermore, permissions were also obtained from Tigray regional health bureau and medical directors of Mekelle hospital and Ayder Compressive Specialized hospital. Each data was kept confidentially using codes and initials instead of names. CD4^+^ and viral load results were attached with the patients’ medical records to be used as follow up and base line data respectively. Study participants who had virological and immunological failures were linked with responsible health professionals for proper management.

## Results

### Socio demographic data of study participants

A total of 260 HIV infected individuals on first line ART regimen were included in the study, of which 151 (58.1%) of them were females. The minimum and maximum age of the participants was 10 and 63 years old, respectively with median age of 39 (IQR: 32–48) years old. One hundred seventeen (45.0%) were married, and 229 (88.1%) of the study participants were infected through sexual contact. The majority 197 (75.8%) of the study participants were attended primary school and above. One hundred four (40%) of them were self-employed with a median income of 1500 birr. Urban dwellers accounted 211 (81.2%) of the participants **(Table 1)**.

**Table 1:** Socio demographic characteristics of HIV infected individuals on ART from January 1, 2008 to March 1,2016 at Mekelle hospital and Ayder Comprehensive Specialized Hospital (n = 260).

Characteristics	Frequency (%)
**Gender**	
Male	109 (41.9)
Female	151 (58.1)
**Age group at initiation of ART (years)**	
10–19	23(8.8)
20–29	54(20.8)
30–39	96(36.9)
40–49	63(24.2)
50–60	24(9.2)
**Age group in median time of 36 months on ART (years)**	
10–19	21 (8.0)
20–29	28 (10.8)
30–39	92 (35.4)
40–49	59 (22.7)
50–63	60 (23.1)
**Residence**	
Rural	49(18.8)
Urban	211(81.2)
**Educational level**	
No formal education	63 (24.2)
Primary	79 (30.4)
Secondary	84 (32.3)
Tertiary	34 (13.1)
**Occupational status**	
Gov’tal employed	40 (15.3)
Self employed	104 (40.0)
Private employed	22 (8.5)
Farmer	15 (5.8)
Unemployed	57 (21.9)
NA*	22 (8.5)
**Marital status**	
Widowed	19 (7.3)
Single	62 (23.8)
Married	117 (45.0)
Divorced	41 (15.8)
NA**	21 (8.1)

Key

* NA- Not applicable for students

** NA-Not applicable for children <18 years old

Study participants were followed for a minimum of 6 and a maximum of 108 months with a median time of 36 (IQR: 24–54) months on ART. At base line 136 (52.3%) of study participants were on clinical stage III/IV. Of the total, 186 (71.5%) of them had at least one or more history of opportunistic infections either at ART initiation or follow up period. Among those who had history of opportunistic infections, 43 (16.5%) of them had oral/esophageal candidiasis (data not shown), 43 (16.5%) TB co-infection and 26 (10.0%) had chronic/acute diarrhea (data not shown). Self-report of participants’ showed that 242 (93.1%) of them were adherent to the treatment. 180 (69.2%) of the study participants were given Tenofovir (TDF) based ART regimen followed by Zidovudine (AZT) (21.2%) (**Table 2**).

**Table 2:** Baseline and follow up characteristics on first line ART from January 1, 2008 to March 1, 2016 at Mekelle hospital and Ayder Comprehensive Specialized Hospital (n = 260).

Characteristics	N° (%)
**Time on ART (months)**	
6–12	19 (7.3)
13–24	56 (21.5)
25–36	71 (27.3)
37–48	45 (17.4)
>48	69 (26.5)
**Route of transmission**	
Mother to child	21 (8.1)
Heterosexual	229 (88.1)
Blood transfusion	10 (3.8)
**Regimen given at base line**	
TDF 3TCEFV/NVP*	180 (69.2)
AZT 3TC NVP /EFV**	55 (21.2)
D4T 3TC NVP /EFV^#^	21 (8.1)
ABC 3TC EFV^##^	4 (1.5)
**WHO staging at base line**	
I/II	124(47.7)
III	107(41.2)
IV	29(11.2)
**Adherence**	
Poor/Fair	18 (6.9)
Good	242 (93.1)
**Reason for non-adherence (n = 18)**	
Side effects	4 (22.2)
Forgot/Social problem	10 (55.6)
Too ill	4 (22.2)
**History of malnourished**	
Yes	83 (31.9)
No	177 (68.1)
Chronic NCDs***	
Yes	12 (4.6)
No	248 (95.4)
**History of OIs**	
Yes	186 (71.5)
No	74 (28.5)
**TB Co-infection**	
Yes	43 (16.5)
No	217 (83.5)
**HIV prophylaxis given**	
Yes	12 (4.6)
No	248 (95.4)
**HBV Co-infection(n = 139)**	
Yes	11 (7.9)
No	128 (92.1)
**HCV Co-infection (n = 64)**	
Yes	2 (3.1)
No	62 (96.9)

Key

* TDF-Tenofovir; 3TC-Lamivudin, EFV-Efavirenz; NVP- Nevirapine

** AZT-Zidovudine

^#^ D4T-Stavudin

^##^ ABC-Abacavir

***chronic NCDs: Chronic non communicable diseases (include Hypertension and Diabetic mellitus)

### Immunological failure and associated risk factors

**Fig 2:**
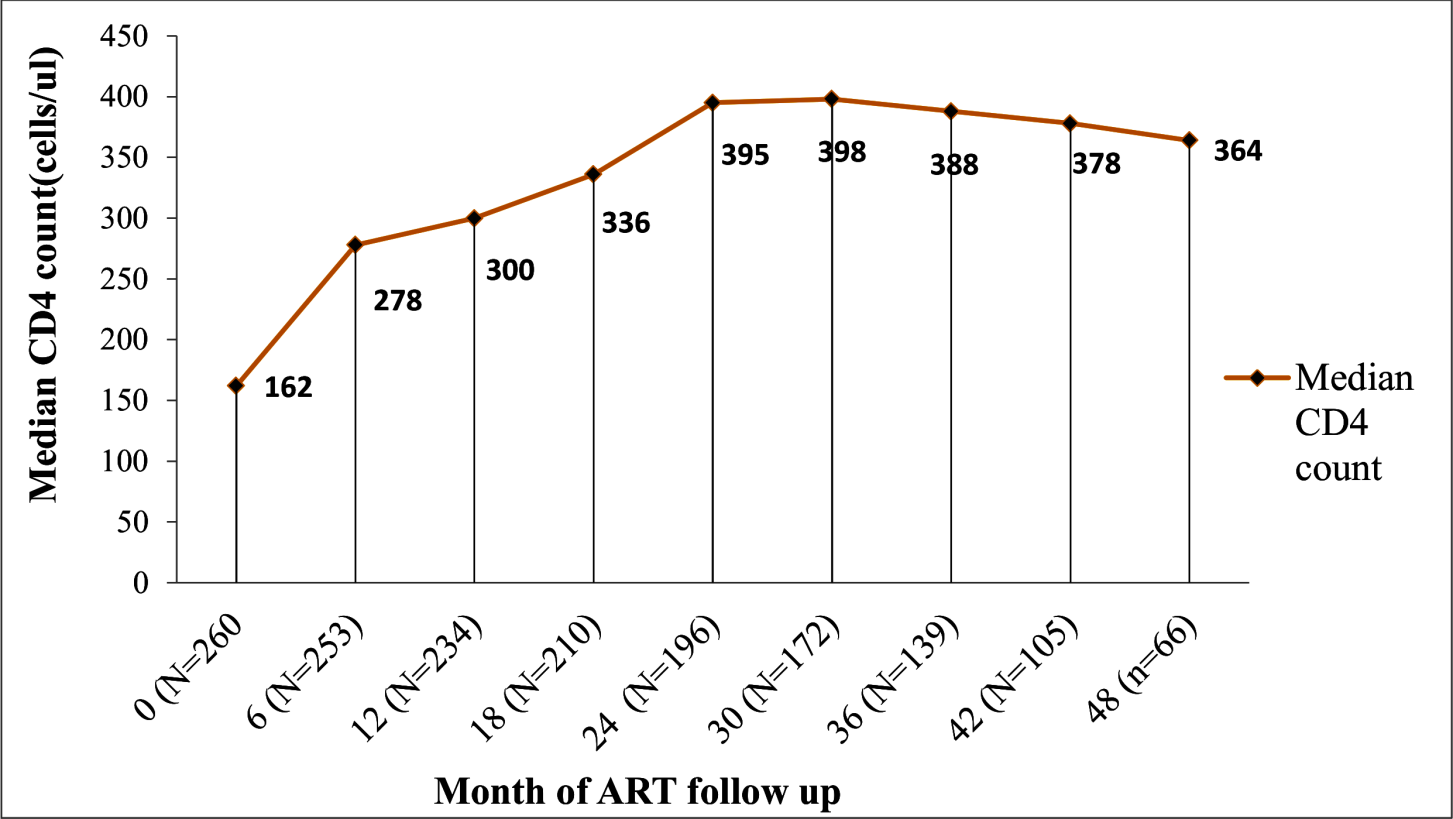
Trends of median CD4^+^ count at different periods of time of study participants on first line ART at Mekelle hospital and Ayder Comprehensive Specialized Hospital.

Study participants were on first line HAART for 6 to 108 months with a median follow up time of 36 months. The baseline median CD4^+^ count was162 cells/uL (IQR: 85–269) and increased by 116 cells/uL, and reached 278 (IQR: 172–413) cells/uL after 6 months of HAART. It further increased to 300, 336, 395 and 398 cells/uL after 12, 18, 24 and 30 months of HAART, respectively (“**Fig 2”**).

In median time of 36 (IQR: 24–54) months of ART follow up, 17 (6.5%) of the study participants had persistent CD4^+^ count below 100 cells/μL which is an indication of immunological failure. The median age among those who had poor immunological recovery was 39 (IQR: 33–43) years. 9/17 (52.9%) and 10/17 (58.8%) of them were males and were given TDF 3TC EFV at base line respectively. In addition, 11/17 (64.7%), 11/17 (64.7%) and 7/17(41.2%) of them have history of malnutrition, opportunistic infections and non-adherence at least once respectively. Furthermore, 11/17 (64.7%) and 14/17 (82.4%) of them had CD4^+^ count < 100 cells/μL and WHO clinical stage III/IV at base line respectively.

Non-adherent study participants were 5.68 times more likely to experience immunological failure (AOR (95% CI) = 5.68 (1.86–19.8), p = 0.021) compared with those who were good adherent by adjusting TB co infections and viral load. Furthermore, it was statistically significant that patients having HIV RNA level ≥ 1000 copies/mL had 12.33 times higher odds of immunological failure (AOR (95% CI) = 12.33(3.06–29.7), p<0.0001) than those who had < 1000 copies/mL by adjusting other factors. Likewise, immunological failure was 3.8 times more likely to occur in TB co-infected individuals (AOR (95% CI) = 3.80 (2.01–12.90), p = 0.003) compared to TB non infected individuals by adjusting adherence and high viral load (**Table 3)**.

**Table 3:** Immunological failure and associated risk factors among HIV infected individuals on ART from January 2008 to March 2016 at Mekelle hospital and Ayder Comprehensive Specialized Hospital(n = 17).

Characteristics	Immunological failure		Binary logistic regression		Multiple logistic regression		
	Yes N (%)	N° N (%)	p- value	OR(95%CI)	p- value	AOR(95%CI)	
**Gender**							
Male	9(8.3)	100 (91.7)					
Female	8(5.3)	143(94.7)	0.35	0.62(0.23–1.67)			
**Age group(years)**							
<40	10(7.1)	131(92.90)					
≥40	7(5.9)	112(94.1	0.69	0.82(0.30–2.22)			
**Age group at initiation of ART(years)**							
<35	6(5.0)	115(95.0)	0.34	1.65(0.59–4.60)			
≥35	11(7.9)	128(92.2)					
**CD4 +count at base line(cells/**μ**L)**							
≤250	16 (10.1)	142(89.9)	NA				
>250	1(0.1)	101(99.9)					
**Time on ART(months)**							
<36	12(11.4)	93(88.6)					
≥36	5(3.2)	150(96.8)	0.014	0.26(0.088–0.78)	0.068	0.32(0.093–1.87)	
**Regimen given at base line**							
TDF 3TC EFV	10(6.1)	155(93.9)	NA				
AZT 3TCNVP	1(2.8)	35(97.2)					
AZT 3TC EFV	3(15.8)	16(84.2)					
Other	3(7.5)	37(92.5)					
**Adherence**							
Fair/poor	7(38.9)	11(61.1)	0.00	14.76(4.72–26.14)	0.021	5.68(1.86–19.8)	
Good	10(4.1)	232(95.9)					
**Viral load(copies/mL) in a median time of 36 months of ART**							
<1000	7(3.0)	223(97)					
≥1000	10(33.3)	20(66.7)	0.00	15.93(5.47–26.38)	0.00	12.33(3.06–29.7)	
**WHO clinical stage at base line**							
I/II	3(2.4)	121(97.6)	NA				
III/IV	14(10.3)	122(89.7)					
**History of malnutrition**							
Yes	11(13.3)	72(86.7)					
No	6(3.4)	171(96.6)	0.005	0.23(0.082–0.65)	0.24	0.47(0.14–1.65)	
**History of opportunistic infections**							
Yes	11(7.7)	132(92.3)					
No	6(5.1)	111(94.9)	0.408	0.65(0.23–1.81)			
Chronic non communicable diseases*							
Yes	2(15.4)	11(84.6)	NA				
No	15(6.5)	232(93.5)					
**TB co-infection**							
Yes	7(16.3)	36(83.7)	0.008	4.03 (1.44–11.26)	0.003	3.80 (2.01–12.90)	
No	10(8.5)	207(91.5)					
**Income(birr)**							
≤1500	7(5.1)	129(94.9)	0.35	1.62(0.60–4.38)			
>1500	10(8.1)	114(91.9)					
**HBV co-infection(n = 139)**							
Yes	3(27.3)	8 (72.7)	NA				
No	8(6.3)	120(93.8)					
**HCV co-infection(n = 64)**							
Yes	1(50)	1(50)	NA				
No	3(4.8)	59(95.2)					

### Virological failure and associated risk factors

Of the 260 participants, 221 (85%) of them had suppressed viral load (Plasma HIV RNA level: <150copies/mL), 9 (3.5%) had 150–999 and 30 (11.5%) of them had high viral load (HIV RNA level ≥1000 copies/mL) after median time of 36 (IQR: 24–54) months on ART.Median viral load and CD4^+^ count among those who had an indication of virological failure (n = 30) was 12,239 (IQR: 2,548–43,622) copies/mL and 202 (IQR: 87–350) cells/μL respectively.

Virological failure was 16.37 times more likely to happen in non-adherent individuals (AOR (95% CI) = 16.37 (4.65–57.68), p <0.001) compared with those who had good treatment adherence by adjusting gender, age and CD4^+^ count. Study participants aged less than 40 years old had 4.43 times more likely to experience virological failure than these who were older than 40 years old (AOR (95%CI) = 4.43 (1.57–12.46), p = 0.005) by adjusting gender, adherence and CD4^+^ count. Likewise, being male was 4.6 times more likely to experience virological failure (AOD (95% CI) = 4.60 (1.72–12.33), p = 0.002) as compared to females by adjusting other factors. Furthermore, individuals having CD4^+^ count <250 cells/μL were 2.81 times more likely to develop virological failure (AOD (95% CI) = 2.81 (1.051–7.51), p = 0.04) as compare with those who have greater CD4^+^ count in a median time of 36 months of ART follow up by adjusting adherence, age and gender (**Table 4**).

**Table 4:** Virological failure and associated risk factors among HIV infected individuals on ART from January 2008 to March 2016 at Mekelle hospital and Ayder Comprehensive Specialized Hospital (n = 30).

Characteristics	Virological Failure		Binary logistic regression		Multiple logistic regression	
	Yes N° (%)	No N° (%)	p-value	OR(95%CI)	p- value	AOR(95%CI)
**Gender**						
Male	20(18.3)	89(81.7)	0.005	3.17(1.4–7.08)	0.002	4.6(1.72–12.33)
Female	10(6.6)	141(93.4)				
**Age group(years)**						
<40	22(15.6)	119(84.4)	0.03	2.57(1.10–6.00)	0.005	4.43(1.57–12.46)
≥40	8(6.7)	111(93.3)				
**Age group at initiation of ART(years)**						
<35	18(14.9)	103(85.1)	0.12	1.85(0.85–4.02)		
≥35	12(8.6)	127(91.4)				
**CD4**^**+**^ **count (cells/**μ**L) at base line**						
≤250	21(13.3)	137(86.7)	0.27	1.58(0.70–3.61)		
>250	9(8.8)	93(91.2)				
**CD4**^**+**^ **count (cells/**μ**L) after median time of 36 months of ART follow up**						
≤250	15(30)	35(70)	0.000	5.57(2.50–12.4)	0.040	2.81(1.051–7.51)
>250	15(7.2)	195(92.9)				
**Time on ART(months)**						
<36	16(15.2)	89(84.8)	0.13	1.81(0.84–3.90)		
≥36	14(9)	141(91)				
**Regimen given at base line**						
TDF 3TC EFV	10(6.1)	155(93.9)	NA			
AZT 3TC NVP	9(25)	27(75)				
AZT 3TC EFV	6(31.6	13(68.4)				
TDF 3TC NVP`	3(20)	12(80)				
Other	2(8)	23(92)				
**Adherence**						
Fair/poor	11(61.1)	7(38.9)	0.00	18.44(6.41–12.08)	0.00	16.37(4.65–57.68)
Good	19(7.9)	223(92.1				
**WHO clinical stage at base line**						
I/II	14(11.3)	110(88.7)	0.91	1.03(0.45–2.05)		
III/IV	16(11.8)	120(88.2)				
**History of malnutrition**						
Yes	14(16.9)	69(83.1)	0.07	2.04(0.95–4.41)		
No	16(9)	161(91)				
**History of opportunistic infections**						
Yes	19(13.3)	124(86.7)	0.33	1.48(0.67–3.24)		
No	11(9.4)	106(90.6)				
**Chronic non communicable disease**						
Yes	5(38.5)	8(61.5)	0.015	5.55(1.69–18.27)	0.07	4.40(0.89–21.81)
No	25(10.1)	222(89.9)				
**TB co-infection**						
Yes	4(9.3)	39(90.7)	NA			
No	26(12)	191(88)				
**HIV prophylaxis given**						
Yes	0(0)	12(100)	NA			
No	30(12.1)	218(87.9)				
**Income(birr)**						
≤1500	13(9.6)	123(90.4)	0.3	0.67(0.31–1.43)		
>1500	17(13.7)	107(86.3)				

## Discussion

In Ethiopia HAART scale up began in 2005 and since then its coverage was 82.8% by 2015 [14]. HAART guideline in Ethiopia is given a combination of two Nucleoside transcriptase inhibitors (NRTI) and one Non-nucleoside transcriptase inhibitor (NNRTI). The first line regimens are Lamivudine (3TC) combined with Stavudine (d4T), zidovudine (AZT) or Tenofovir (TDF), and either nevirapine (NVP) or efavirenz (EFV) (Table 2). However, d4T is discontinued due to its known long term mitochondrial toxicity and substituted either by AZT or TDF. For anemic patients TDF substitutes AZT and for tuberculosis patients treated with Rifampicin EFV replaces NVP.

This study described immunological and virological failure and associated risk factors among HIV infected individuals on first-line regimen from 6 months to 9 years duration on ART. Twenty nine (11.2%) of the study participants were under the category of clinical WHO stage IV at ART initiation. After 6 months of ART, WHO stage IV cases had decreased to 13 (5.0%) and fall to 1 (0. 4%) after median time of 36 months ART follow up. This was similar to a study conducted in Surat, India [17].

Majority (93.1%) of the study participants had good adherence (≥ 95%) to treatment, similar value to one study conducted in Colombia 92% [18]. However, our result was higher than the reports from Addis Ababa 78.5% [19], Gonder (82.7% [20] and 85.8% [21], southern Ethiopia 81.8% [22], and New Guinea 82.4% [23]. On the other hand, our finding was lower when compared to Addis Ababa 97.7% [24] and Jimma 100% [25]. These variations in patient adherences may be possibly related with psychosocial support, stigma, lack of commitment to take medications, not feeling well, fear of side effects and being busy or forgetting [12, 26, 27].

The median baseline CD4^+^ counts of the study participants’ was 162 cells/μL. This was comparable with the studies conducted in southern Ethiopia 156 cell/μL [22] and Kenya 152 cells/μL [28], but higher than that of Addis Ababa 115 cells/μL [19]. Studies from Addis Ababa 177 cells/μL [24], Jimma 191 cells/μL [25], and Liberia 238 cell/μL [29] have reported higher median CD4^+^ count than our study. This variation may be explained with differences in time of initiation of ART in patients infected by HIV for long period of time. Since long duration of HIV infection without initiation of ART favors viral replication which in turn leads to lower CD4^+^ count.

Immunological failure was found to be 6.5% in a median time of 36 month of ART follow up. Our finding was similar with a study conducted in Liberia 5.1% [29]. However, it was much lower than the findings in other parts of Ethiopia: southern Ethiopia 11.5% [22], Jimma 9.8% [25], Addis Ababa 15.7% [19] and 15% [24], Gonder 15.1% [20], and other studies outside Ethiopia: Kenya 64.4% [28], Tanzania 25% [30], China 18.4% [31], Colombia 14% [18], Thailand 33.5% [32], and Nepal 35% [33].

This variation in immunological failure may be related with the WHO guideline which varies overtime. For example, in our study immunological failure was defined based on the 2016 WHO guide line, as fall of CD4^+^ count below 250 cells/μL following clinical failure, or persistent CD4^+^ count below 100 cells/μL [12]. However, the aforementioned researchers [19,20,22,25,28,30,31] defined immunological failure based on previous WHO guidelines as fall of CD4^+^ count to baseline or below sever immune suppression (CD4^+^ count< 200 cells/μL) [31], 50% fall from on-treatment peak [19,24,22,31] or fall by 30% from on treatment peak value [28,30], failure to achieve CD4^+^ count > 350 cells/μL despite virological suppression [32], CD4^+^ count < 350 cells/μL [33]; in addition to persistent CD4^+^ count below 100 cells/μL. Besides, higher immunological failure in other studies may be attributed to the higher non-adherence rate [19, 20, 22], compared with our study.

Non-adherent individuals were 5.68 times more likely to experience immunological failures than those who had good adherence (p = 0.021). This is in tandem with the study conducted in Gonder [20], southern Ethiopia [28], Colombia [18] and France [34]. Possibly this explanation may be due to the fact that poor adherence allow more viral replication which in turn increases infection of more CD4^+^ cells and ultimately depletion of their number.

Immunological failure was 3.8 times more likely to occur in TB co-infected individuals compared to TB non infected individuals (p = 0.003). This was similar with reports from southern Ethiopia [28], Gonder [20, 35] and Nigeria [36]. This indicated that TB infection impairs cellular immune responses through M.tuberculosis-induced apoptosis of CD4 cells which subsequently lead to depletion of CD4^+^ cells and results in immunological failure [37].

Study participants with HIV RNA level ≥ 1000 copies/mL showed 12.33 times more likely to experience immunological failure (p <0.001) in a median time of 36 months of ART follow up as compared with those who have HIV RNA level less than 1000 copies/mL. Similar results have been reported from India [38], Nepal [33], Thailand [39], London [40], and USA [41].

The overall virological failure (viral load ≥1000 copies/mL) in this study was seen in 30 (11.5%) study participants. This was similar with a study conducted in Gonder 11.8% [21], Addis Ababa 15% [21], New Guinea 13.7% [23], Uganda 14.5% [42], China 13.4% [31], Thailand 9.6% [32], Nepal 9.9% [33] and Cambodia 12.9% [43]. However, our result was higher than findings from studies conducted in Jimma 5.3% [25] and Burkina Faso 7.5% [44]. This variation may be due to difference in interventions for optimization of patient’s adherence and type of regimen given. For example, protease inhibitors were used as first line regimen in Burkina Faso [44] and nutritional support was given for the study participants [25] which were different from our setting.

Unlike to our result, higher virological failure was reported from Kenya 23.7% [45] and 24.6% [46], Liberia 47% [29], Zimbabwe 30.6% [47], Tanzania 57.1% [30], 32% [48], Cameroon 20.6% [49], Ghana 16.7% [50], Togo 51.6% [51], Colombia 20.9% [18], India 69.6% [38] and Peru 24% [52]. This difference may be due to lower treatment adherence compared with our study [48, 52]. The other possible reason may due to differences in definition of virological failure, where plasma viral load ≥400 copies/mL was used as cut-off point for virological failure [30, 45, 46] and inclusion criteria used. For example, the study participants included in those studies were clinically/immunologically failed [29, 39] and perinatally infected [51], and had lower median age (9.5 years old) [49] and shorter median time on ART (7.8 months) [50].

Non-adherence was strongly associated with virological failure (AOR = 16.37, p < 0.001), this was comparable with a studies conducted in Gonder [21], Uganda [53], New Guinea [23], Colombia [18], Kenya [46], Tanzania [48,54], Peru [52] and Thailand [32]. This is because low level of antiretroviral in the body owing to the non-adherence is not sufficient to suppress viral replication, hence leads to detection of HIV RNA level in the blood [53].

Likewise, being male was 4.6 fold risk to develop virological failure (p = 0.002) than females and this was lined with a study conducted in Burkina Faso [44], Swaziland [55] and Nigeria [56]. Similarly higher virological failure, but statistically non-significant was reported from Zimbabwe [47, 57], Kenya [28], Nepal [33], result of worldwide systemic review [58] and Colombia [18]. Possible explanation for virological failure among males has been speculated due to their low health–seeking behavior [26, 44, 56] as compared to females.

Virological failure was 4.43 times more likely to happen in individuals aged less than 40 years old (AOR(95% CI) = 4.43(1.57–12.46), p = 0.005). This was supported by several studies conducted in Kenya [28, 47], South Africa [59], Burkina Faso [44], Swaziland [55], result of systemic review in Sub Saharan Africa [26], India [38], Thailand [32] and Johns Hopkins-North Carolina University [60]. The possible explanation for this may be due to difference in maturity or lifestyle stability or may be due to work load and hence may forget to take medication properly. However, this needs further study.

Low CD4^+^ count may reflect viral replication due to treatment interruption or resistance [61]. This appears consistent relationship between current CD4^+^ count and odds of virological failure. Those who had CD4^+^ count <250 cells/μL were 2.81 times more likely to have virological failure (p = 0.040) in a median time of 36 months of ART as compared with those whose CD4^+^ count was ≥ 250 cells/μL. This association was similar with studies conducted in Tanzania [48], Swaziland [55] and Peru [52]. Though not statistically significant, studies conducted from Burkina Faso [44] and Boston [62] have supported our result.

## Limitation of the study

Our study was not without limitation. One may argue that taking consecutive viral load measurements would be a better strategy to determine the virological failure than a single measurement. However, in Ethiopia and mainly in the study area, patients on HAART were followed using CD4+ count and WHO clinical stages. Hence we were unable to get data on viral load from the patient’s chart and that could be our limitation of the study. Due to the budget limitations, we only did a single viral load measurement during the collection time. Therefore, researches using consecutive viral load measurements are needed to have a representative data on the virological failures in the country.

## Conclusion

After a median time of 36 months on ART, virological and immunological failure in the study area were (11.5%) and 17(6.5%), respectively. Non-adherence, aged < 40 years old, having CD4^+^ count < 250 cells/μL in a median time of 36 months on ART, and male gender were predictors for virological failure. Similarly, non-adherence, TB co-infection and HIV RNA level ≥1000 copies/mL were associated with immunological failure. Hence our result highlights the need for more commitment and effort from all stake holders on these predictors so as to sustain the long term efficacy of HAART there by achieving the ambitious target of 2020.

## Supporting information

S1 Table Questionnaire. (DOCX)

S2 Table Raw patients’ data. (SAV)
