# Supplementary material for: Virological and immunological failure of HAART and associated risk factors among adults and adolescents in the Tigray region of Northern Ethiopia
Source: PLoS One. 2018 May 1;13(5):e0196259. doi: 10.1371/journal.pone.0196259 (PMC5929526; doi:10.1371/journal.pone.0196259)
Supplement: S1 Table — (DOCX) [file pone.0196259.s001.docx]

| 1. **Participants identification**   Code No_________________, MRN_________________,Unique ART No:__________________  phone no: ______________________,Sex:_________ , Hospital_______________ |
| --- |
| 1. **Socio demographic characteristics** |
| 1. Age(years ) : <1 year (months): |
| 1. Month of ART follow up: |
| 1. Current Regimen given: **A**. First line regimen: _______________ **B.** Second line regimen:­­­­­_____________ |
| 1. Route of transmission: A. Vertical mother to child B. Sexual C. Contaminated Blood D. Needle injury E. Others(specify):_____________________ |
| 1. Marital status: **A**. Widowed **B.** Single **C.** Married **D**. Divorced |
| 1. Educational level : **A**. Unable to read and write **B**. 1-6 grades **C**. 7-12 grade **D**. College level and above |
| 1. Occupational status: **A**. House wife **B.** Governmental employed **C.** Self employed   **D**. Private employee **E.** Farmer **F**. Unemployed **G.** Student **I.** Child |
| 1. **Socio economic status** |
| 1. Income(birr): |
| 1. Residence: **A**. Rural  **B**. Urban |
| 1. **Information related to HIV infection and ART** |
| 1. Do you smoke? 2. Yes (if yes how many cigarettes per day): _______________________________**B**. No |
| 1. Did you have exposure of multiple sexual partners/unsafe sex during ART follow up:   **A**. Yes (**if yes go to no 3**) **B**. No |
| 1. Did you use condom. **A.** yes **B**. No |
| 1. Do you drink alcohol**: A**. yes (How often=______________________) B. No |
| 1. Do you have fever: A. Yes B. No |
| 1. Do you have known chronic disease: **A.** Yes (If any specify):___________________ **B**. No |
| 1. Is the participant pregnant (For females only)? A. Yes B. No |
| 1. Is the participant breastfeeding (For females only)? A. Yes B. No |
| 1. Primary Caregiver for children (go to no 10 if age is greater than 14):   **A**. Biological Mother **B**. Father  **C**. Aunt **D**. Uncle **E**. Grandmother/Father **F**. others |
| 1. Current Psychiatric illnesses: **A.** Yes  **B**. No |
| 1. Current nutritional status (adults’and adolescents): **A**. Not malnourished **B**. Mild **C**. Moderate **D**. Severe malnutrition |
| 1. Current nutritional status( for children): 2. Normal **B**. Mild **C**. Moderate acute malnutrition **D**. Severe acute malnutrition |
| 1. Current TB status : **A**. Positive **B.** Negative |
| 1. Current TB prophylaxis /TB Treatment given: **A** Yes (Specify the drug given)________________________**B**.No |
| 1. Current presence of opportunistic infection/cancer: **A**. Yes :( specify type):_______________________**B.** No |
| 1. Current opportunistic infection/cancer prophylaxis or treatment given:   **A**. Yes (Mention type of drug):_____________________________________**B.**  No |
| 1. Any current regimen/drug change? **A.** Yes (If yes specify reason and type ):____________________­­­­­­­________**B**. No |
| 1. Current WHO Clinical stage:________ |
| 1. Current ART Adherence (%): |
| 1. Any non adherence (Poor/fair): A. Yes (If yes specify reason):___________________________B. No |
| 1. Current HBV status: **A**. Positive **B.** Negative C. Not done |
| 1. Current HCV status: **A.** Positive **B**. Negative C. Not done |
| 1. Current Hemoglobin level (if done): A. ______g/dl B. Not done |
| 1. Current Liver function test result (if done): A. Normal B. Abnormal C. Not done |
| 1. CD4 count and percentage: 2. Result=_____________________cells/uL: _________________%. |
| 1. Viral load result(copies/ml): |

| 1. **Base line and follow up characteristics (**From medical records and charts**)** |
| --- |
| 1. Entry date: |
| 1. Age during ART initiation (years)_________________,<1 year (in months)____________, |
| 1. Date confirmed HIV+: |
| 1. Date of ART initiation(dd/mm/yyy): |
| 1. First line regimen given at base line (Adult ): |
| 1. If Pediatric, first line regimen given at base line: |
| 1. Nutritional status (Normal /Mild /Moderate/ Severe/ ) 2. At base line______B. At 6 month: _____C. At 12 months: ____D. At 18 months: ____E. At 24 month=______ 3. At 30 months: ____G. At 36 months: ____H. At 42 months: __I. At 48 months: _____ J. Other (if any):_______ |
| 1. History of Nutritional supplements dispense: 2. At base line B. At 6 month C. At 12 months D. At 18 months E. At 24 month F. At 30 months 3. At 36 months H. At 42 months I. At 48 months J. Other (if any) ___________K. Not given |
| 1. History of TB at baseline (when initiating ART): A. Yes B. No C. Not done |
| 1. History of TB at follow up: A. Yes (specify month of ART follow up) ____________________B. No |
| 1. History of taking TB prophylaxis /TB Treatment at base line: A. Yes (Specify drug)______________B. No |
| 1. History of taking TB prophylaxis /TB Treatment at follow up: 2. No B. Yes (specify month of ART follow up and type of drug):__________________________ |
| 1. HBV status at base line: A. Positive B. Negative C. Not done |
| 1. HBV status at follow up: A. Positive (mention month of ART follow up if any)_____________ B. Negative C. Not done |
| 1. HCV status at base line: A. Positive B. Negative C. Not done |
| 1. HCV status at follow up: A. Positive (mention month of ART follow up if any)______________   B. Negative C. Not done |
| 1. History of opportunistic infections/cancers at baseline: A. Yes (specify type)_______________B. No |
| 1. History of opportunistic infections/cancers at follow up:   A. Yes (specify month of ART follow up and type):___________________________________ B. No |
| 1. History of taking prophylaxis /treatment for opportunistic infections at base line: 2. Yes (specify type of drug):_____________________________________________________B. No |
| 1. History of taking prophylaxis /treatment for opportunistic infections at follow up: 2. Yes (specify type of drug and month of ART follow up):_______________________________B. No |
| 1. Liver function test (Normal/abnormal):   A. At base line: ___B. At 6 month: ___C. At 12 months: __ D. At 18 months: ___E. 24 month=_____  F. At 30 months: ___G. At 36 months: ___H. At 42 months: ___ I. At 48 months: ___ J. Other (if any):__ |
| 1. Hemoglobin level:    - - 1. At base line: ___B. At 6 month: ___C. At 12 months: __ D. At 18 months: ____E. 24 month=____ 2. At 30 months: ___G. At 36 months: ___H. At 42 months: ___I. At 48 months: ___ J. specify if any):__ |
| 1. WHO clinical stage: 2. At base line: ___B. At 6 month: ___C. At 12 months: __ D. At 18 months: ____E. 24 month=_____   F.At 30 months: ___G. At 36 months: ___H. At 42 months: ___I. At 48 months: ___ J. Other (if any):__ |
| 1. Any ART regimen/drug change at follow up: A. Yes (**if yes go to no 25, 26 and 27**) B. No |
| 1. Mention the new regimen/drug given:________________ |
| 1. Month of ART follow up during new regimen/drug given:___________ |
| 1. Reason for Regimen/drugs change :______________________ |
| 1. ART adherence (%): 2. At 6 month: ____B. At 12 months: ___C. At 18 months: ___D. 24 month=____E. At 30 months: ____ 3. At 36 months: ____G. At 42 months: ____H. At 48 months: ____I. Other (if any):____ |
| 1. If any non adherence(Fair /poor): 2. Specify reason : _____________________,B. Month of ART follow up:_____________ |
| 1. Route of transmission: A. Vertical mother to child B. Sexual C. Contaminated Blood D. Needle injury E. Others(specify):_____________________ |
| 1. Smoke behavior at base line or follow up: A. Yes: B. No |
| 1. History of multiple sexual partners/unsafe sex: A. Yes B. No |
| 1. History of drinking alcohol at base line or follow up: A. yes: B. No. |
| 1. Know chronic disease at base line: A. Yes (If any specify):__________________________B. No |
| 1. Know chronic disease at follow up: A. Yes (If any specify month of ART follow up):_________B. No |
| 1. Primary Caregiver for children ( for age less than 14 years old): 2. Biological Mother B. Father C. Aunt D. Uncle E. Grandmother/Father F. Others:___________ |
| 1. CD4 cell count (cells/uL):   A. At base line: ___B. At 6 month: ___C. At 12 months: __ D. At 18 months: ___E. 24 month=_____  F. At 30 months: ___G. At 36 months: ___H. At 42 months: ___ I. At 48 months: ___ J. Specify if any:__ |
| 1. CD4 (percentage):   A. At base line: ___B. At 6 month: ___C. At 12 months: __ D. At 18 months: ___E. 24 month=_____  F. At 30 months: ___G. At 36 months: ___H. At 42 months: ___ I. At 48 months: ___ J. Specify if any:__ |
| 1. Presence of Psychiatric illnesses at base line: A. Yes B. No |
| 1. Presence of Psychiatric illnesses at follow up: A. Yes (if yes how many times)______________B. No |
| 1. History of lost follow up: A. Yes (If any mention month of ART follow up) ________________ B. No |
| 1. History of Previous pregnancy before initiation of ART (for females only): A. Yes (If yes go to no 42) B. No |
| 1. History of taking HIV prophylaxis during pregnancy for PMTCT:   A. Yes (if yes, mention the drug):________________________________________B. No |
